# Twins and their singleton siblings differ in language, cognition, and social-emotional development

**DOI:** 10.1093/chidev/aacaf029

**Published:** 2026-03-24

**Authors:** Emily Wood, Anna Brown, Sophie von Stumm

**Affiliations:** Department of Child & Adolescent Psychiatry, Institute of Psychiatry, Psychology and Neuroscience, King's College London, London, United Kingdom; School of Psychology and Behavioural Sciences, University of Northampton, Northampton, United Kingdom; Department of Education, University of York, York, United Kingdom

**Keywords:** twins, language, cognition, social-emotional, individual differences

## Abstract

This study compared the language, cognition, and social-emotional development between twins and their younger singleton siblings at the ages 2, 3, 4, and 7 years. Data were collected 1996–2004 from 851 sibling trios from the Twins Early Development Study (TEDS; 50.2% female; 93.2% White British). Distributions were comparable between twins and singletons across domains, indicative of mean rather than variance discrepancies. Twins performed worse than singletons in language, cognition, and social-emotional development across ages, with small to moderate effect sizes (*d* = 0.17–0.62). An exception was language at age 7 years, when twins exceeded their singleton siblings (*d* = −0.35). Overall, twins and their singleton siblings differed in development across domains and their differences in language reversed during childhood.

In developmental science, monozygotic twins, who are genetic clones and share 100% of their genes, are often compared with dizygotic twins, who share, on average, 50% of their segregating genes. These comparisons allow inferring the extent to which children's developmental differences can be attributed to genetic versus environmental influences. Twin studies have made numerous pivotal contributions to our understanding of children's developmental differences (Plomin et al., 2016). Yet, the method of twin pair comparisons and corresponding interpretations of findings are not without criticisms (Evans & Martin, 2000). Here, we explore one of these criticisms: The longstanding claim that twins' development in early childhood differs substantially from that of singletons (i.e., birth of only one child during a single delivery; Thorpe, 2006; Voracek & Haubner, 2008).

Differences in twins' and singletons' development have been observed in several domains. Perhaps best known is the delay in language development that twins show relative to singletons. On average, twins reach the same language developmental milestones 2 to 8 months later than singletons (Thorpe, 2006). Finding that twins and singletons differ in development suggests that twins and singletons also differ in their early life experiences, which has three important implications.

First, systematic developmental differences between twins and singletons could challenge the generalizability of twin findings to singleton populations. Twin studies that elucidate the origin of children's differences assume that developmental experiences are comparable across twins and singletons. If twins and singletons have different early life experiences, twin study estimates of genetic and environmental influences on child development may not be applicable to and valid for singleton populations.

Second, finding that twins are disadvantaged in development relative to singletons or vice versa would call for better support for the disadvantaged population, to prevent and ameliorate developmental delays. For example, twins with delayed language development in early life may perform worse in primary school than singletons with typical language development because language skills predict school readiness and academic achievement (Bleses et al., 2016; Pace et al., 2019). In this case, early intervention could help mitigate twins’ risk of struggling in school, before maladaptive learning behaviors have manifested (e.g., low motivation, avoidance). Such interventions would ensure that twins achieve optimal learning outcomes akin to singletons.

Third, understanding the causes of twins' and singletons' differences in development can provide insights into the environmental factors that affect development. For example, it may be that twins and singletons are exposed to different quantities and qualities of speech or that they differ in their access to learning resources such as books and educational toys. By learning about twins' and singletons' differences in their early life, we can better appraise the value of individual characteristics of the language environment for healthy language development. These insights would benefit our understanding of development for both twin and singleton populations.

Prior studies in this area relied mostly on between-family study designs that compared twins' psychological development to that of unrelated singletons (e.g., Christensen et al., 2006; Pulkkinen et al., 2003; Webbink et al., 2008). However, such comparisons are confounded by unmeasured genetic and environmental factors that vary across families (Dick et al., 2000). Twins and singletons that are not born and raised in the same families may differ in development due to a vast range of factors that differ between families, independent of being twins or singletons. Perhaps not surprisingly then, prior between-family studies produced inconsistent findings (DiLalla, 2006; Nan et al., 2013; Pulkkinen et al., 2003; Webbink et al., 2008). For example, one study showed that twins' delay in language development relative to singletons diminished by the age of 12 months (Nan et al., 2013). Yet, another study showed that the delay in language development reduced much later, by the age of 10 years (Webbink et al., 2008). Analyses of data from twins and their singleton siblings allow controlling for family-level confounding (Susser et al., 2010). Comparisons between twins and singletons within the same family index the effects of being a twin or singleton on development, independent of between-family effects.

For this study, we used a within-family design to test whether twins and their younger singleton siblings differed in their language, cognition, and social-emotional development, which allows accounting for between-family level confounding. Because our data were longitudinal, we could observe the degree of developmental changes within subjects and between twins and their singleton siblings. Twins' and singletons' differences were assessed using reliable psychometric scales across core domains of development. To the best of our knowledge, this study is the first in the literature to use a within-family study design that charts twins' and singletons' differences in language, cognition, and social-emotional development from age 2 through to 7 years.

## Differences in parenting twins versus singletons

Parents play an important role in their children's development, providing physical (e.g., food, shelter), emotional (e.g., love, emotional security), cognitive (i.e., play and stimulation), and social support (i.e., manners, values; Jeong et al., 2021; World Health Organization, 2004). There are several ways that parenting twins may differ from parenting singletons. First, twins must share their parents' attention compared with singleton births that enjoy parents' undivided attention (Conway et al., 1980). This is especially true for singleton first-borns, who, on average, are afforded the greatest time and attention from their parents, as they have no siblings to compete with for parenting resources (Bornstein, 2005). Parents also devote more time and attention to single newborns relative to their older siblings, who have increased self-sufficiency; yet, this is not possible with twin newborns that are likely equally demanding in care (Kidwell, 1981). The need to divide their attention will reduce the extent to which twin parents interact and engage with each of their twin children (Lytton et al., 1977). For example, twin children are, on average, talked to and held less often than singleton children (Holditch-Davis et al., 1999).

Second, raising twins is more stressful for parents than raising singleton children. Twins' parents report parenting to be more difficult and stress-inducing than singletons' parents, and they describe on average fewer experiences of pleasure when parenting (Olivennes et al., 2005). Additionally, mothers of twin children report lower wellbeing, and higher stress, exhaustion, and depression compared with mothers of singletons (Andrade et al., 2014). These and similar strains have been found to affect the speech that parents direct at their twins, which includes shorter, less sophisticated utterances than speech directed at singletons (Lytton et al., 1977; Trombetta et al., 2019). Twin parents' speech is also often more directive than responsive toward their children, and activities like joint book reading can be less engaging for the twin than singleton children (Trombetta et al., 2019). These and other differences in language experiences between twins and singletons may produce subsequently differences in language development.

Third, raising twins is economically more costly than raising singletons: Twins' parents must purchase duplicate essentials for their twins, including splitting hand-me-downs from older siblings. Childcare costs for twin children during the early years can be twice as high as for singletons or siblings of two different ages (McKay, 2010). Plus, it is often not possible for both parents to return to work following a twin birth, as a result of which household incomes may be more limited (Campbell et al., 2004). Financial constraints influence the parenting of singleton births, because parents' increased stress adversely affects their responses to children's developmental needs (Puff & Renk, 2014). Financial constraints are likely even greater after twin than singleton births and thus, parenting twins may be more stressful than parenting singletons.

One prior study found little evidence for differences in parenting twins as compared to singleton children (Mönkediek et al., 2020). When mothers' parenting of twins was compared with that of their older singleton siblings, no differences were observed in emotional warmth, inconsistent parenting, or strict control in a sample of 980 German families (Mönkediek et al., 2020). However, twins received somewhat less negative communication and psychological control from their parents than their singleton siblings (Mönkediek et al., 2020). The effect sizes were small, suggesting that any differences in the parenting of twins and their nontwin siblings were minimal.

## Socialization experiences of twins versus singletons

Twins and singletons vary in their socialization experiences, which may contribute to differences in development. First, twins share the company of their co-twin from conception; they start life by sharing a womb, and monozygotic twins, originating from the same fertilized egg, often even share the same placenta, including in some cases even the same amniotic sac. Twins later continue to share much of their postnatal environment. They are accustomed to competing for resources, for example having triadic conversations with caregivers (Barton & Strosberg, 1997) and sharing clothing and toys (Segal & Knafo-Noam, 2020). This differs from singletons and non-twin siblings, whose gaps in age mean that they have different needs and are often more accepting of differential treatment, reducing competition between them (Fortuna et al., 2010). The unique experience of sharing a co-twin from birth may shape twins' development in ways that are not directly comparable to that of singletons. Being used to playing with a child who is genetically and phenotypically similar to themselves, twins may become reluctant to socialize with other, un-related children (DiLalla & Caraway, 2004). Alternatively, twins may develop advanced social interaction skills through their experiences with their co-twin, which would help them to engage and cooperate with others (DiLalla, 2006). Forming healthy relationships with peers is important for children's wellbeing and development (Bryan et al., 2013). Unlike parents, who provide guidance and structure for their children, peers offer experiences in collaboration, conflict, and social belonging (Hepler, 1997).

Second, being a twin prompts more attention from peers and the public than singletons typically receive, particularly for twins of high physical resemblance (Danby & Thorpe, 2006). Identical twins are often identified as a pair rather than individually. They are called by each other's name by friends and family and compared with one another in both appearance and personality (Määttä et al., 2016). These and related experiences that are unique to twins may affect their social-emotional development. For some twins, others' emphasis of their alikeness could encourage forming deep connections, with twins sharing hobbies and friends, and even developing their own secret language (Hayashi et al., 2014). Yet, twins may also struggle to achieve independence from each other and a positive, individual identity that is separate from their role as twin (Akerman, 2003), which could affect their social-emotional development (Potterton et al., 2022). Meanwhile, singletons are more likely to be treated as independent from their siblings and thus are better able to develop a positive sense of identity (Noble et al., 2017).

## Twins' and singletons' developmental differences

Above, we described how twins' parenting and socialization experiences during the early years are likely to differ from those of singletons. These differences may translate into developmental differences between twins and singletons, which we describe here. We focus on three key developmental domains: language, cognition, and social-emotional development. These domains are fundamental to healthy child development, and they are prevalen foci of developmental research. They also have pervasive, long-term influences on life outcomes, including educational attainment and mental wellbeing (Dale et al., 2023; Deary et al., 2007; Payton et al., 2008). Children's development of language entails understanding and producing language, by growing lexicon and grammar, verbal concept formation, and verbal reasoning (Fenson et al., 2023; Wechsler, 1949). Cognition broadly refers to the ability to reason, plan, think, and solve problems, which are central to the cognitive constructs of intelligence, theory of mind, and executive function (e.g., Deary, 2012; Diamond, 2013). Social-emotional development includes understanding and managing one's emotions, learning to express and respond to emotions, and using emotions to relate to others and develop healthy relationships with them (Malti & Noam, 2016). Social-emotional development is often, including in the current study, inferred from measures of peer and conduct problems, hyperactivity, prosocial behavior, and emotional symptoms (cf. Strengths and Difficulties Questionnaire, Goodman, 1997; Hobbs et al., 2007).

1. Delay in language

The magnitude of twins' language delay, if given as a time difference between twins and singletons reaching the same language development milestones, is thought to be 2 to 8 months behind singletons (Thorpe, 2006). This estimate was based on a review of seven studies on twin-singleton differences in language ability that were published between 1932 and 2003 (Thorpe, 2006). The review's study inclusion criteria were not described. All the studies relied on between-family comparisons and did not conduct within-family analyses (i.e., comparing twins to their siblings). To the best of our knowledge, no meta-analysis or more up-to-date review of the twin-singleton differences in language development has been published. A recent meta-analysis concluded that data were also too limited to draw conclusions about differences between twins' and singletons' linguistic environments (Trombetta et al., 2019).

Some prior studies addressed the persistence of twins' language delay in comparison to singletons. A within-families comparison of a Dutch sample, taken from the Netherlands Twin Registry, found that twins outperformed their singleton siblings in reading and language tests from ages 7 to 12 years (*N* = 762 sibling trios; de Zeeuw et al., 2012). A between-families comparison of a nationwide sample of Dutch school pupils showed that twins' (*N* = 3,894) language test scores were lower than those of unrelated singleton school pupils (*N* = 188,208) until the age of 10 years, when the twin-singleton difference became nonsignificant (Webbink et al., 2008). However, another study reported that twins' developmental delay in communication skills, when compared with unrelated singletons, diminished already by the age of 12 months in a smaller sample of 100 twins (Nan et al., 2013). Although a language delay in twins relative to singletons is widely assumed, evidence for its magnitude and persistence are inconsistent. This inconsistency may result from differences in sample size and test instruments, and variation in the use of within- and between-family comparisons.

Delay in cognition

There is evidence to suggest twins' exhibit delays in cognition relative to singletons. A meta-analysis that compared cognition in over 15,000 twin pairs and 1.5 million singleton births concluded that twins scored on average 4.1 IQ points lower than singletons in childhood and adolescence (Voracek & Haubner, 2008). Only two of the included studies sampled twins and singletons from the same families (Ronalds et al., 2005; Webbink et al., 2008). The first tested 236 twins and 9,832 singletons born in Aberdeen in the 1950s, some of which were from the same families (N not reported; Ronalds et al., 2005). The study found that twins scored on average 5.3 IQ points lower at age 7, and even 6 IQ points lower at age 9 years than their singleton siblings (Ronalds et al., 2005). The second study compared trios of 196 Dutch twins and their singleton siblings when they had reached adulthood; here, no difference in IQ was observed (Webbink et al., 2008).

Some studies have explored the persistence of twins' delay in cognition (Eriksen et al., 2012; Posthuma et al., 2000; Webbink et al., 2008). For example, a study of 3,894 twins and 188,208 unrelated singletons found that they differed on average by less than one IQ point between the ages of 8 to 12 years (Webbink et al., 2008). Further studies confirmed that differences in twins' and singletons' cognition are likely minimal in later childhood and adolescence (Christensen et al., 2006; Hjern et al., 2012). For instance, a within-families study of 5,364 twins and their 3,085 singleton siblings, born in Sweden from 1973 to 1981, found no difference in grade point average at age 14 to 15 years, and in IQ test scores at age 18 to 19 years (Hjern et al., 2012). Another study of 1,653 Norwegian sibling trios found male twins to score on average 1.7 IQ points lower than their singleton brothers at age 18 to 20 years old (Eriksen et al., 2012). Despite the small inconsistencies in findings, the evidence suggests overall that twins' and singletons' differences in cognition decrease or diminish over the childhood years.

An important issue in comparing twins and singletons' developmental outcomes are differences between countries and generations. Findings from Denmark and the Netherlands suggested that in recent generations, twins and singletons differed in cognition to a lesser extent than previously (e.g., Christensen et al., 2006; Ronalds et al., 2005; Webbink et al., 2008; see Voracek & Haubner, 2008). Improvements in the healthcare and social support for twin mothers in WEIRD (Western, Educated, Industrialized, Rich, and Democratic; Henrich et al., 2010) countries may have helped to decrease differences in twins' and singletons' cognition (Christensen et al., 2006; Voracek & Haubner, 2008). By contrast, 826 Nigerian twins, who were born in the 1990s and had no access to high-quality health and social care, scored on average 4.2 IQ points lower than 280 unrelated singletons in adolescence (Hur & Lynn, 2013). This difference is much larger than has been observed in WEIRD samples of that generation and age (Christensen et al., 2006; Webbink et al., 2008). While these findings suggest that later generations of twins born in WEIRD countries may differ less in cognition from singletons, not all evidence aligns with this interpretation (e.g., Eriksen et al., 2012).

To conclude, evidence on the magnitude and persistence of twins' and singletons' differences in cognition is inconclusive, likely due to variations in sample size (i.e., statistical power), location (WEIRD versus nonWEIRD countries), and birth year, as well as differences in test instrument (e.g., Raven's Standard Progressive Matrices Plus versus the Wechsler Adult Intelligence Scale) and within- or between-family comparisons. That said, the general consensus is that twins and singletons differ in cognition during the early years, and that this difference diminishes over the course of childhood.

Social-emotional development

To the best of our knowledge, no meta-analytic evidence is currently available on twin-singleton differences in ­social-emotional development. Earlier studies in this area produced mixed results. Some reported that being a twin leads to more favorable social-emotional development than being a singleton. For example, in a Finnish cohort study of 1,874 twins aged 11 to 12 years and 23,200 of their singleton boy classmates, twin boys were perceived more often as socially active, which was reflected by their leadership, popularity, and interactions with others (Pulkkinen et al., 2003). Likewise, twin girls were rated more often as socially active and constructive than singleton girls (Pulkkinen et al., 2003). However, corresponding effect sizes were small (i.e., Cohen's *d* ranged from 0.18 to 0.22 for girls, and 0.14 to 0.24 for boys). Similarly, a between-family Japanese study of 106 pairs of twins (mean age 5 years) and 86 pairs of singleton siblings (younger sibling mean age 4 years, older sibling mean age 7 years) who were unrelated to the twins, found that twins scored higher than singletons on tests of prosocial behavior, though the difference was not statistically significant (Nozaki et al., 2012). From these studies, one might infer that being a twin versus a singleton has a small positive effect on social-emotional development.

Other studies reported deficits in twins' social-emotional development relative to singletons. For example, in observations of peer-play between 5-year-old twins (*N* = 62) and unrelated singletons (*N* = 143), twins engaged less often in prosocial behaviors than singletons but displayed no differences in aggressive behavior (DiLalla, 2006). When comparing the same twins (*N* = 62) and unrelated singletons (*N* = 77) at age 10 to 15 years, parents rated twins as more aggressive than singleton children, and twin-singleton differences in prosocial behavior were no longer evident (DiLalla, 2006). Effect sizes were small; yet the findings suggest that being a twin versus a singleton can have a negative effect on social-emotional development. As it stands, existing literature on the effect of being a twin versus a singleton on social-emotional development is inconclusive.

## The current study

We capitalized on data from twins and their younger singleton siblings who were part of the TEDS. Twins and their younger siblings were assessed on their language, cognition, and social-emotional development at their respective ages of 2, 3, 4, and 7 years. We addressed two core research questions: (a) Do twins relative to their siblings show delays in language, cognition, and social-emotional development? And (b) do these twin-singleton differences in development change across assessment ages? Our preregistered hypotheses were as follows:

Hypothesis 1: On account of the existing literature on twins' and singletons' language differences (cf. de Zeeuw et al., 2012; Thorpe, 2006; Webbink et al., 2008), we predicted that twins would score lower on language tests than their younger singleton siblings at ages 2 through to 7 years. We made no predictions about the magnitude and persistence of twins' language delay, because of inconclusive previous findings.

Hypothesis 2: We predicted that twins would score lower on tests of cognition than their younger siblings, but that this difference would decrease by age 7 years. This is due to the evidence, albeit inconsistent, that twins' and singletons' differences in cognition decrease during childhood (Christensen et al., 2006; Hjern et al., 2012; Webbink et al., 2008).

For differences in twins' and singletons' social-emotional development, we did not formulate specific hypotheses due to the mixed findings of previous research in this area (DiLalla, 2006; Nozaki et al., 2012; Pulkkinen et al., 2003).

## Method

### Sample

Data came from TEDS, a longitudinal cohort study that recruited over 15,000 families with twins born in England and Wales between 1994 and 1996. The TEDS recruitment process and sample are described in detail elsewhere (e.g., Lockhart et al., 2023). A subsample of 851 families who had a younger sibling born within a maximum of six years after the twins' birth served for the preregistered analyses of the current study. Twins' assessments took place between 1996 and 2004; their siblings' assessments took place between 1997 and 2004. We included families for whom data was available for both twins and their younger sibling for at least one of the developmental measures (language, cognition, or social-emotional development) collected during at least one assessment age (2, 3, 4, or 7 years). We excluded families who reported severe medical problems during gestation, birth, or the perinatal period, or that either a twin or their sibling had learning difficulties or problems with hearing, speech, or eyesight. Following exclusions, our sample comprised of 2,553 individuals: 851 twin pairs (1,702 twin individuals), and 851 younger siblings. Of both twins and siblings, 50.2% were female and 93.2% were White British. Of the twins, 49.8% were female and 61.3% were dizygotic, of which 42.5% were opposite sex. Of the singleton siblings, 50.9% were female (for details see Table S1). Sample demographic characteristics were representative of the UK population at first data collection. We compared the socioeconomic status (SES) of our analysis sample to that of the overall TEDS population (*z*-score comparisons). SES was inferred from parents' education, occupation, and mothers' age at first birth (not necessarily the twins' birth). In our analysis sample, the mean was 0.04, with SD = 1.00 (for details see Table S2), which is minimally higher than that of the overall sample (*M* = 0, SD = 1).

## Measures

### Developmental outcomes

When their participating children were 2, 3, and 4 years old, parents completed booklets that were mailed to them via post with questions relating to their children's development. At age 7, data were collected directly from each child during telephone calls; booklets with accompanying stimuli were posted to each participating household.

### Language

Language was measured at age 2, 3, and 4 using questions adapted from the MacArthur-Bates Communicative Development Inventories (MCDI; Fenson et al., 2023). The questions were designed for parents to answer on behalf of their children.

At age 2, parents completed a 100-item measurement of their children's vocabulary size (“Please tick the words you have heard your child use”), 5 items on children's word use (e.g., “Does your child ever talk about past events or people who are not present?”), and a 13 item measure of sentence complexity (“For each pair of sentences below, tick the one that sounds most like the way your child talks at the moment”). At age 3, the same measures were used, adjusted to a difficulty appropriate for 3-year-old children. At age 4, vocabulary and grammar tests included a 48-item measure of expressive vocabulary (“Please tick the words from the list below that you have heard your child say”) and a 14-item measure of abstract language (“Does your child talk about the order of events by using words like ‘before’ and ‘after’?”).

At age 7, vocabulary was assessed using the 18-item vocabulary test from the Wechsler Intelligence Scale for Children (WISC; Wechsler, 1949) adapted for administration via telephone (“Tell me what each word means”). Verbal concept formation and verbal abstract reasoning were tested using a 13-item WISC Similarities test, also adapted for telephone delivery (“I am going to say two words and ask you how they are alike”).

### Cognition

At the ages 2, 3, and 4 years, cognition was assessed with the Parent Report of Children's Abilities (PARCA; Oliver et al., 2002; Saudino et al., 1998), which was adapted by TEDS for parents to administer to their children. The validity of the PARCA has been previously demonstrated; for example, PARCA scores correlate *r* = .55 with scores on the Mental Development Index (Saudino et al., 1998) and *r* = .54 with the McCarthy Scales of Children's Abilities nonverbal composite (McCarthy, 1972; Oliver et al., 2002). At age 2, PARCA tests included a 4-item drawing, 8-item matching, 1-item folding, 7-item copying, and 4-item brick building tasks, as well as a 26-item conceptual knowledge test completed by the parent (e.g., “Does your child recognize him or herself when looking in the mirror?”). Items administered at age 3 were similar but adjusted in difficulty to suit 3-year-old children. In addition, a 16-item odd-one-out task was included. At age 4, the odd-one-out and drawing tasks were repeated at a more advanced level, as were the conceptual knowledge questions. A draw-a-man task was included, as was a 12-item puzzle task.

At age 7, cognition was tested using a 9-item conceptual grouping task (McCarthy, 1972) and a 21-item picture completion task (from the Weschler Intelligence Scale for Children; Wechsler, 1949) administered to the participant via telephone.

### Social-emotional development

Social-emotional development was assessed through parent-ratings on the Strengths and Difficulties Questionnaire (SDQ; Goodman, 1997) or an SDQ-comparable scale, which used standard measures taken from the Revised Rutter Parent Scale for Preschool children (RRPSPC; Hogg et al., 1997), the Children's Behavior Questionnaire (Rutter, 1967), and the SDQ (Goodman, 1997). The SDQ includes 25 items, with 5 items respectively measuring prosocial behavior (“tries to be fair in games”), conduct (“fights with other children”), peer problems (“not much liked by other children”), emotional problems (“is worried, worries about many things”), and hyperactivity (“has poor concentration, or short attention span”). The SDQ-comparable scale includes very similar, though fewer, items: At age 2, the scale consisted of 4 items for conduct problems (“destroys own or other's belongings”), 2 items for emotional problems (“appears miserable, unhappy, tearful or distressed”), 3 items for hyperactivity (“restless […] doesn’t keep still”), 3 items for peer problems (“not much liked by other children”), and 5 items for prosocial behavior (“volunteers to help around the house or garden”). At age 3, the scale consisted of the same items from age 2, with an extra item for emotional problems (“often complains of stomachaches, headaches or feeling sick”). At age 4 and 7, the original SDQ scale was used (see Tables S3 and S4 for further details of all items used).

## Covariates

### Socioeconomic status

SES was included in our statistical analyses as a covariate, as it was expected to covary with the effect of being a twin versus a singleton on developmental outcomes. SES was indexed by a composite from mothers' and fathers' educational qualification level, mothers' and fathers' occupational classification and mothers' age at birth of first child (not necessarily the twins'). Family SES was reported at first contact and when the twins were 7 years old. Corresponding family SES data for the younger siblings at the same ages was not collected. As family SES scores at first contact and at age 7 were significantly, highly correlated (*r* = .76, *p* ≤ .001), scores were averaged across both assessment time points to form a single score, reflecting the average family SES during the twins' and singletons' early years.

### Number of children in the household

The number of children living in the household was included as a covariate in our statistical analysis to account for its effect on children's early life environments (e.g., Downey, 2001). At first contact, parents reported the number of children living in the home in addition to their twins. Then, at twins' ages 2, 3, 4, and 7 years, parents were asked to report any new children that had joined to live in the household. Parents did not provide this information during the siblings' data collection, so we were unable to create a time-specific covariate to account for the age at which new children were introduced to the household for both the twins and their younger siblings. Instead, we summed all reports of new children in the household to estimate the total number of children living in the household during data collection.

### Zygosity

Zygosity was added as a covariate to all models. Zygosity is unlikely to influence the differences between twins' and singletons' language and cognition because the rearing environments that affect these domains are thought to be equal for MZ and DZ twin pairs (Mitchell et al., 2007). However, the equal environments assumption for MZ and DZ twins may be less likely to hold for rearing environments that affect social-emotional development (DiLalla, 2006; Määttä et al., 2016; Nozaki et al., 2012). For consistency, we added zygosity as a covariate across all models. Twins' zygosity was measured at first contact, age 3, and age 4 years, through questionnaires that focused on twins' physical differences.

### Statistical analysis

Our analyses were preregistered (https://osf.io/gbm9r/files/q98ge) and conducted using the statistical programme R version 4.4.0 (R Core Team, 2023) and the R package lme4 (Bates et al., 2015). Missing data were handled by list wise deletion, which is the standard procedure for modeling with lme4. We *z*-transformed all measures; for language and cognition, we took the respective measures' mean to create composite scores. Both the language and cognition measures are recognized valid measures (Fenson et al., 2023; Oliver et al, 2002; Saudino et al., 1998; Wechsler, 1949) and are commonly used as additive scores (e.g., Dale et al., 2015; Hayiou-Thomas et al., 2012). The *z*-transformations were conducted within each age of assessment (e.g., mean = 0 and SD = 1 in cognition at age 2, 3, 4, and 7 years) across twins and their singleton siblings.

We accounted for the nested structure of our data with multilevel linear models (Aarts et al., 2014). We built models through an iterative, stepwise approach, whereby fixed and random effect terms were introduced sequentially to the model. After each term's introduction, the model was compared with the previous one, to test whether model fit had improved. Random or fixed effects which did not improve model fit were dropped from the model. First, we added subject- and family-varying intercepts to the models, to account for the relatedness of the data within each group (Model 1). Second, we specified random slopes for age (i.e., 2, 3, 4, and 7 years) to account for individual differences in the effect of age on each outcome (Model 2). Third, we added the covariates sex, SES, zygosity, and number of children in the household to account for their potential confounding effects (Model 3). Fourth, we included a fixed effect for being a twin versus a singleton and age (i.e., 2, 3, 4, and 7 years; Model 4). Finally, an interaction term was added to account for the possibility that the effect of being a twin versus a singleton on development varied by age (Model 5).

Model fit was estimated using AIC (Akaike Information Criterion). Except for language, hyperactivity, and peer problems, models that included the random intercepts and slopes, the covariates sex, SES, zygosity, and number of children in the household, and the fixed effects of being a twin versus a singleton and age proved the best fit. For language, hyperactivity, and peer problems, an interaction term between being a twin versus a singleton and age explained additional variance and significantly affected model fit. A summary of the model comparisons can be found in Tables S5–S11.

To interpret the results of the multilevel models, we first evaluated unstandardized model estimates with 95% confidence intervals. Then, we conducted post-hoc pairwise comparisons using the estimated marginal means from each model to establish the extent of twins' and singletons' differences in each domain. For models without an interaction term, we tested the significance and effect size of the difference between twins' and singletons' mean scores. For models with an interaction term, we also observed how differences in twins' and singletons' scores varied by age. A Bonferroni correction was applied to account for the increased likelihood of Type I errors when conducting multiple comparisons. We conducted 4 comparisons (twins' and singletons' scores at ages 2, 3, 4, and 7) at a 0.05 significance level, giving a corrected significance level of 0.05/4 = 0.0125. We derived Cohen's *d* from each comparison and used this value, as well as the *p*-value, to interpret the results.

## Results

**Figure 1:**
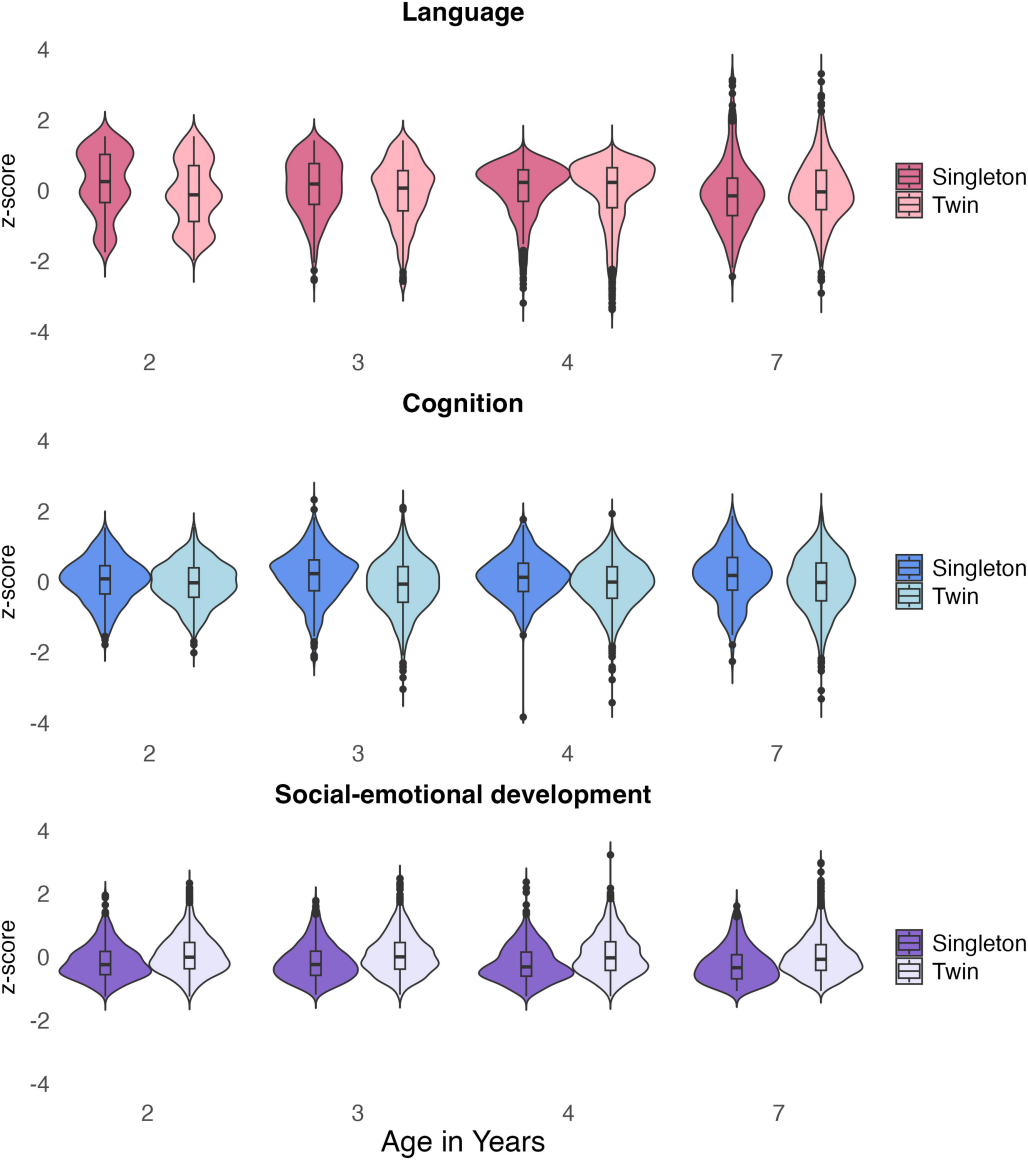
Twins' and singletons' language, cognition, and social-emotional development from age 2 to 7 years. The violins represent the distribution of the data for each group, at each age. The boxplots within each violin depict the interquartile range—the median, upper, and lower quartile. The black dots represent outliers. The social-emotional violin plot was computed using a composite of all social-emotional measures, with the prosocial behavior measure reverse-scored.

Descriptive statistics for all continuous study variables can be found in Table S2. Most variables were normally distributed, except for peer problems and emotional problems (i.e., lower scores were more frequent), as is typical for these measures. Figure 1 shows the distribution of twins' and singletons' scores in language, cognition, and social-emotional development. Overall, distributions were comparable between twins and singletons across domains, indicative of mean rather than variance discrepancies between sibling types.

### Language

**Figure 2:**
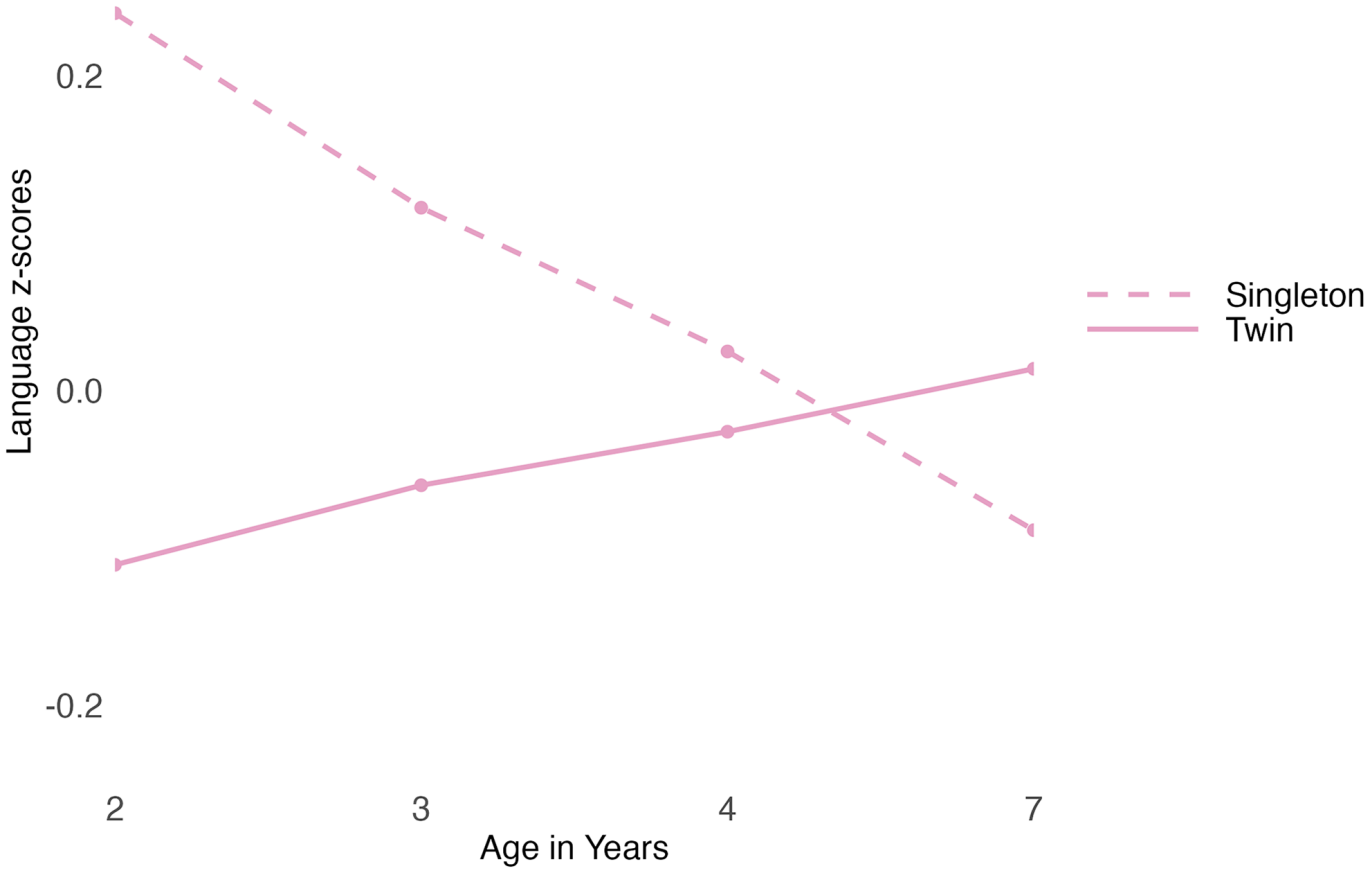
Language of twins and their younger singleton siblings at ages 2 to 7 years. The graph shows the mean *z*-transformed language scores of singletons and twins at each age. The graph has not been adjusted for covariate effects.

Model comparisons suggested that the interaction term between being a twin versus a singleton and age explained significant unique variance in language (see Table S5 for details) and achieved a parsimonious model. Thus, we found that the effect of being a twin versus a singleton on language varied by age (Figure 2; full model results are shown in Table S12).

Pairwise comparisons showed that singletons outperformed twins at ages 2, 3, and 4 years in language (*d* ranged from 0.12 to 0.41, *p* ranged from.002 to < .001), with their difference being biggest at age 2 years and decreasing at age 3 and 4 years. By age 7 years twins' language exceeded that of singletons (*d* = −0.35, *p* = <.001; see Figure 2 and Table S13 for details). This suggests twins' language is delayed compared with singletons' during the early years, but this delay reverses by age 7 years.

### Cognition

**Figure 3:**
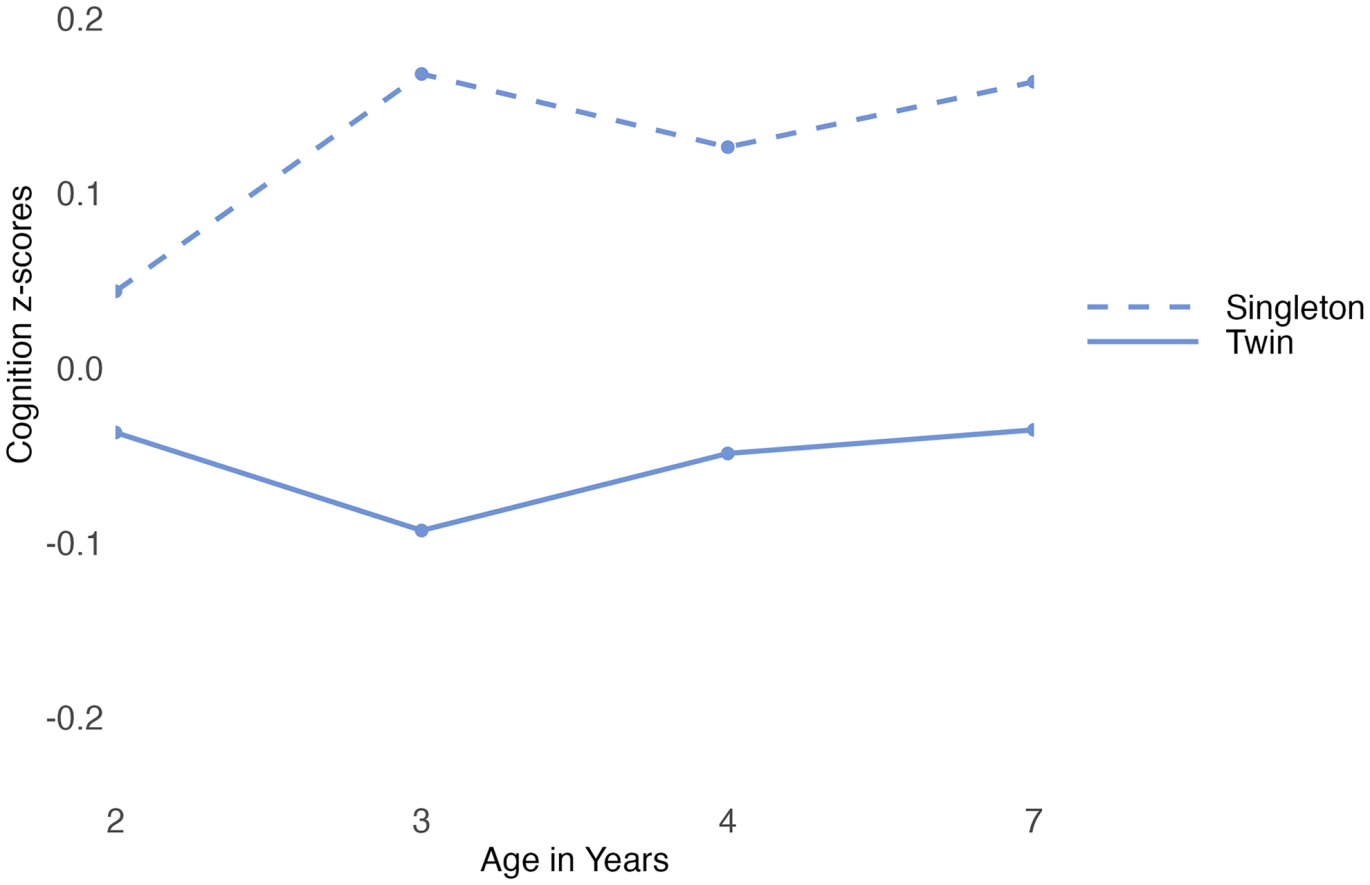
Cognition of twins and their younger singleton siblings at ages 2 to 7 years. The graph shows the mean *z*-transformed cognition scores of singletons and twins at each age. The graph has not been adjusted for covariate effects.

For cognition, the interaction term between being a twin versus a singleton and age did not explain significant unique variance. Therefore, the most parsimonious model for cognition included being a twin versus a singleton and age as independent fixed effects (i.e., no interaction; see Table S6 for details). This suggested that the effect of being a twin versus a singleton on cognition was consistent across ages. Singletons scored higher than twins on cognition at all ages (*B* = −0.17, 95% CI = −0.20 to −0.14; see Table S12 for details). A pairwise comparison indicated this difference to be significant (*d* = 0.34, *p* = <.001, see Table S13 for details). Thus, twins' cognition appears delayed compared with singletons' from age 2 years until at least age 7 years (see Figure 3).

### Social-emotional development

The interaction term between being a twin versus a singleton and age did not explain unique variance in prosocial behavior, conduct problems, and emotional problems. Therefore, the most parsimonious model for these outcomes included being a twin versus a singleton and age as independent terms (see Tables S7, S8, and S11 for model comparisons). Thus, the effect of being a twin versus a singleton on prosocial behavior, conduct problems, and emotional problems was consistent across the ages. Twins scored higher than singletons on conduct problems (*B* = 0.21, 95% CI = 0.16–0.26) and emotional problems (*B* = 0.42, 95% CI = 0.37–0.47), and lower on prosocial behavior (*B* = −0.12, 95% CI = −0.17 to −0.07). Pairwise comparisons showed differences in twins' and singletons' conduct problems (*d* = −0.30, *p* ≤ .001), emotional problems (*d* = −0.55, *p* ≤ .001), and prosocial behavior (*d* = 0.17, *p* ≤ .001) to be significant.

**Figure 4:**
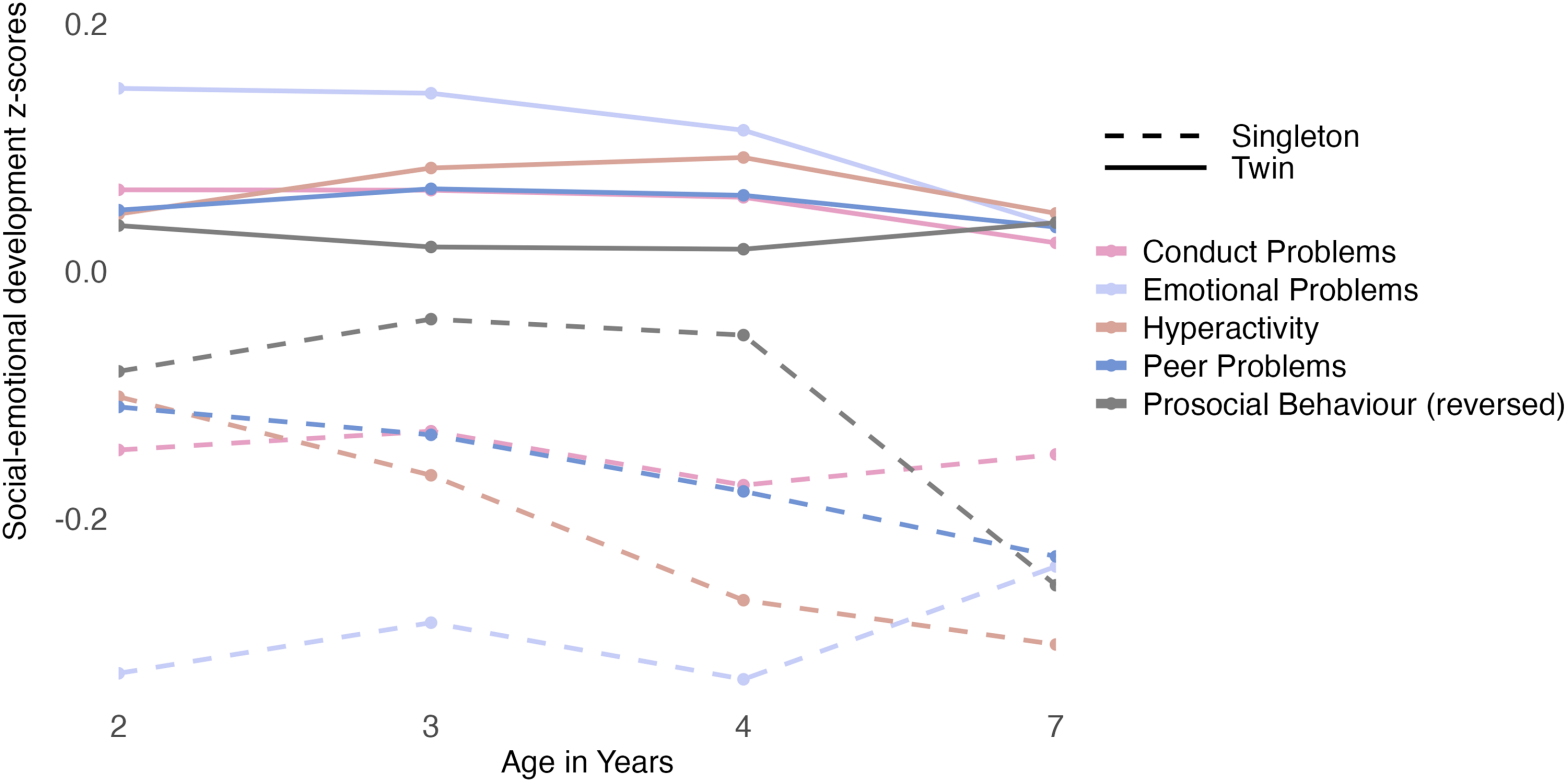
Social-emotional development of twin pairs and their younger singleton siblings at ages 2 to 7 years. The graph shows the mean *z*-transformed social-emotional development scores of singletons and twins at each age. The graph has not been adjusted for covariate effects. The prosocial behavior measure has been reverse scored for the purpose of this graph.

Finally, model comparisons suggested that the interaction term between being a twin versus a singleton and age explained unique variance in hyperactivity and peer problems (see Tables S9 and S10). Thus, the effect of being a twin versus a singleton on hyperactivity and peer problems varied with age. Pairwise comparisons showed that twins scored higher than singletons on hyperactivity (*d* ranged from −0.33 to −0.62, *p* ≤ .001 in all cases) and peer problems (*d* ranged from −0.19 to −0.43, *p* ≤ .001 in all cases). These differences increased with age, so twins' and singletons' differences in hyperactivity and peer problems were largest at age 7 years (see Figure 4 and Table S13 for details). Overall, twins showed more social-emotional problem behaviors than singletons and scored lower on tests of prosocial behavior. For hyperactivity and peer problems, twins' and singletons' differences significantly widened with age.

## Discussion

We investigated whether being a twin, compared with being a singleton, predicts differences in language, cognition, and social-emotional development at ages 2, 3, 4, and 7 when comparing twins and singletons from the same families. Our results make three contributions to the literature.

First, we found that twins and singletons differ in language, cognition, and social-emotional development from ages 2 to 7 years, with small to medium effect sizes. Our findings corroborate existing evidence of differences in twins' and singletons' development (DiLalla, 2006; Thorpe, 2006; Voracek & Haubner, 2008). They also extend the previous literature, showing that differences occur across the developmental domains of language, cognition, and social-emotional development, which have rarely been studied together. From these findings, we can infer that differences exist in twins' and singletons' early environments which influence multiple domains of their development in small, but significant ways.

Second, we observed the persistence of twins' and singletons' developmental differences to vary by domain. By age 7, twins no longer exhibit a language delay when compared with singletons, but their differences in cognition and social-emotional development remained and, in some cases, even widened. These findings suggest that differences in twins' and singletons' environments do not affect each developmental domain equally. Rather, their influence on cognition and social-emotional development is more pervasive than on language. Consequently, twins may reap greater benefits from receiving support for cognition and social-­emotional development than for language, which improves to meet singletons' abilities without intervention.

Third, our findings indicate differences in twins' and singletons' language, cognition, and social-emotional development can be observed when comparing twins and singletons from the same families. This finding addresses a gap in the literature as just a few prior studies compared twins' and their singleton siblings' development, focusing on cognition and language (de Zeeuw et al., 2012; Eriksen et al., 2012; Hjern et al., 2012; Ronalds et al., 2005; Webbink et al., 2008) but omitting social-emotional development. Family-level differences, which may confound differences in twins' and singletons' development, are difficult to control for when comparing unrelated twins and singletons. We speculate that family-level confounding is a key reason for the inconsistency in earlier findings on twins' and singletons' developmental differences. Our research suggests that differences in twins' and singletons' language, cognition, and social-emotional development are evident and systematic when family-level differences are controlled for.

Notably, our analyses demonstrated mean differences between twins' and singletons' development, yet the variability in development that we observed within each group was comparable. This finding implies that twins range just as much as singletons in language, cognition, and social-emotional development, even though they achieve on average lower scores. Results based on analyses of variance in twin samples are therefore likely to be generalizable to populations of unrelated individuals. It follows that findings from behavioral genetic investigations that leveraged twin data can be applied to understanding unrelated children's differences in development. We also note that in our analyses, twins' developmental delays were unlikely to reflect vulnerabilities at the extremes, because relative to their overall number, as many twins as singletons performed one or more Standard Deviations above or below the mean.

Although our research has strengths, including a large sample of twins and singletons from the same families and longitudinal measures of multiple developmental outcomes, it is not without limitations. We address three key concerns here.

First, whilst this research benefited from a repeated measures design, data on twins' and singletons' development was only available at the ages of 2, 3, 4, and 7 years. This means we were unable to observe how twins' and singletons' developmental differences progressed between the ages of 4 and 7 years, and beyond age 7 into adolescence. Consequently, our study could not address at what age between 4- and 7-years twins catch up to singletons in language, and for how long twins' and singletons' cognition and social-emotional differences persist. The prior literature offers inconsistent answers to these questions (e.g., Nan et al., 2013; Webbink et al., 2008). In particular, additional research on twins' and singletons' developmental differences from ages 3 to 6 years is needed.

Second, whilst we could compare twins and singletons from the same families, only data from younger siblings were available. Including older siblings in our analyses would have allowed us to examine how our findings apply to first-born singletons, who research suggests are advantaged in development relative to younger siblings. For example, one study found that twins' older singleton siblings outperformed them in arithmetic, reading, and language, but there was no difference in these domains between the twins and their younger siblings (de Zeeuw et al., 2012). Our study would have also benefitted from controlling for the age difference between twins and their singleton siblings, as sibling density can affect development. For instance, siblings with bigger age differences have been found to perform better on tests of verbal ability, math’s, and reading than siblings who are closer to each other in age (Buckles & Munnich, 2012; Powell & Steelman, 1990). Unfortunately, data on participants' dates of birth were removed from the TEDS database for confidentiality purposes, so we were unable to calculate the age difference between the twins and their singleton siblings. Another related issue is that we controlled in our analyses for some confounding effects but not for others. While within-family comparison studies like ours adjust for confounding due to shared genetic and environmental factors, nonshared environmental experiences that differ between twins and between sibling trios are not controlled. For example, one of two twins may have attended out-of-home childcare during the early years, or their younger sibling may have attended childcare while the twins did not. Such differences in early life childcare experiences, which were not recorded in TEDS, may have contributed to within-family differences in development. Finally, although we accounted for sex differences between individuals, we did not control for sex-based within-family differences, such as whether twins were opposite- or same-sex, and whether their sex matched their younger siblings. Modeling the complex structure of sex matches and mismatches between opposite-sex twins and their siblings would have required including multiple interaction terms that were beyond the scope of the current analyses. In supplementary analyses (not preregistered), we found that controlling for the sex match or mismatch in sibling trios in families with same-sex twins had negligible effects on the results (for details, see Table S14). Consequently, it is unlikely that twins' and siblings' sex differences affected our findings.

Third, we could not investigate whether twins' and singletons' differences varied across generations or countries. Our sample allows us to draw conclusions about 90's born British twins and singletons, being a UK-representative sample, but our findings are not generalizable to samples from other countries or generations. Some evidence suggests that differences in twins' and singletons' cognition have decreased across generations in WEIRD but not in nonWEIRD countries (Christensen et al., 2006; Hur & Lynn, 2013; Voracek & Haubner, 2008). Future research is needed to evaluate generational and country effects on twins' and their singleton siblings' developmental differences.

### Implications

With the limitations outlined above in mind, we propose that our findings have three key implications. First, finding that twins' and singletons' language, cognition, and social-emotional development differ in small but significant ways is relevant when applying twin study heritability estimates to singleton populations. Researchers should be aware of the mean differences in twins' and singletons' development, even if they are of modest effect size, and practice caution when translating findings from twin samples to singleton populations. Second, our findings suggest that twins may benefit from additional rearing support during their early years to avoid them falling behind singletons in development. Supporting twins early can help circumvent the accumulation of developmental disadvantages over time, for example reducing the risk of learning difficulties in school or the manifestation of maladaptive behaviors. Indeed, early interventions in language, cognition, and social-emotional development have been shown to be effective in improving children's life chances (Camilli et al., 2010; Roberts & Kaiser, 2011). Third, our findings open avenues for new research into twins' and singletons' differences in early life environments, including parenting, access to resources, and socialization experiences. Such research could offer clues about the salient correlates behind healthy child development.

## Conclusion

In comparison with their younger singleton siblings, we found that twins performed worse in language, cognition, and ­social-emotional development during their early years. The effect sizes of these twin-singleton differences in development were small to medium. Twins' disadvantages in cognition and social-emotional development persisted from age 2 to 7 years and even widened in some cases (i.e., hyperactivity and peer problems). Yet, twins caught up to and exceeded singletons in language by the age of 7 years. We conclude that researchers should practice caution when applying findings from twin samples to singleton populations. We also propose that twins may be offered additional rearing support during the early years to ameliorate their disadvantages in cognition and social-emotional development relative to singletons.

## Supplementary Material

aacaf029_Supplementary_Data

## Data Availability

The data necessary to reproduce the analyses presented here are available upon request to the TEDS steering committee (https://www.teds.ac.uk/researchers/teds-data-access-policy) and the materials are publicly accessible (www.teds.ac.uk/datadictionary/home.htm). Our analysis code is not publicly accessible. This study was preregistered on the Open Science Framework (https://osf.io/q98ge).
