# Supplementary material for: Twins and their singleton siblings differ in language, cognition, and social-emotional development
Source: Child Dev. 2026 Mar 24;97(3):649–61. doi: 10.1093/chidev/aacaf029 (PMC13176922; doi:10.1093/chidev/aacaf029)
Supplement: aacaf029_Supplementary_Data [file aacaf029_supplementary_data.docx]

**Supplementary Materials**

**Twins and their singleton siblings differ in language, cognition, and social-emotional development**

See here for the preregistration of this research: <https://osf.io/q98ge>

Table of Contents

[Deviations from the preregistration 2](#_Toc195017602)

[Descriptive statistics 3](#_Toc195017603)

[**Table S1: Descriptive statistics of sample** 3](#_Toc195017604)

[**Table S2: Descriptive statistics of measures** 4](#_Toc195017605)

[Model comparisons 7](#_Toc195017606)

[**Table S5: Language** 7](#_Toc195017607)

[**Table S6: Cognition** 8](#_Toc195017608)

[**Table S7: Conduct Problems** 9](#_Toc195017609)

[**Table S8: Emotional Problems** 10](#_Toc195017610)

[**Table S9: Hyperactivity** 11](#_Toc195017611)

[**Table S10: Peer Problems** 12](#_Toc195017612)

[**Table S11: Prosocial Behaviour** 13](#_Toc195017613)

[Table S12: Final model estimates 14](#_Toc195017614)

[Table S13: Post hoc pairwise comparisons 16](#_Toc195017615)

## **Deviations from the preregistration**

There were minor and major deviations from the preregistration. First, we decided to add zygosity as a covariate to all models for consistency, rather than only those for language and cognition as preregistered. Second, our preregistration referred to a sample of 720 participants, which we assumed prior to data access. After data access, we found data were available for 2,553 participants. Finally, we proposed statistical analyses that used multi-level linear regressions and ANCOVAs. Yet, we decided after gaining data access but prior to analysis that multi-level models and post-hoc pairwise comparisons were more appropriate. This change was recorded in an updated preregistration.

## **Descriptive statistics**

### **Table S1: Descriptive statistics of sample**

|  | Total N = 2553 |
| --- | --- |
| Twinship | N (%) |
| Twin | 1702 (66.7) |
| Singleton | 851 (33.3) |
| Sex |  |
| Male (all) | 1272 (49.8) |
| Female (all) | 1281 (50.2) |
| Twin male | 854 (50.2) |
| Twin female | 848 (49.8) |
| Singleton male | 418 (49.1) |
| Singleton female | 433 (50.9) |
| Zygosity (twin pairs) |  |
| MZ | 329 (38.7) |
| DZ | 522 (61.3) |
| DZ twin sex difference |  |
| Opposite sex | 222 (42.5) |
| Same sex | 300 (57.5) |
| Ethnicity |  |
| Asian | 60 (2.3) |
| Black | 18 (0.7) |
| Mixed race | 81 (3.2) |
| White | 2379 (93.2) |
| Other | 9 (0.4) |
| NA | 6 (0.2) |

### **Table S2: Descriptive statistics of measures**

| Variable | N | Mean | SD | Min | Max | Skew | Kurtosis |
| --- | --- | --- | --- | --- | --- | --- | --- |
| SES | 2553 | 0.04 | 1.00 | -2.29 | 2.28 | -0.01 | -0.92 |
| Household children | 2553 | 1.42 | 1.17 | 0.00 | 7.00 | 1.29 | 2.43 |
| Language | 2510 | 0.02 | 0.87 | -3.37 | 3.31 | -0.51 | 0.29 |
| Cognition | 2524 | 0.01 | 0.68 | -3.43 | 2.33 | -0.29 | 0.29 |
| Prosocial behaviour | 2538 | 0.01 | 1.00 | -4.10 | 1.54 | -0.50 | -0.29 |
| Peer problems | 2532 | -0.02 | 0.99 | -1.12 | 5.69 | 1.26 | 1.94 |
| Hyperactivity | 2540 | -0.02 | 0.99 | -1.64 | 2.62 | 0.52 | -0.37 |
| Emotional problems | 2539 | -0.02 | 0.98 | -1.16 | 4.77 | 1.24 | 1.42 |
| Conduct problems | 2538 | -0.02 | 0.99 | -1.48 | 4.77 | 0.86 | 0.88 |

Note: All outcome measures are composites of z-transformed scores, giving a mean of ~0 and SD of ~1. Household children refers to children joining the household in addition to the twins.

### **Table S3: SDQ-comparable scale items**

| **Trait measured**  (Intercorrelation range with SDQ scales) | **Items used (ages 2 and 3 years)** |
| --- | --- |
| **Conduct**  (0.28 – 0.51) | Irritable, quick to fly off the handle |
|  | Is disobedient |
|  | Tells lies |
|  | Bullies other children |
| **Emotional problems**  (0.20 – 0.38) | Is worried, worries about many things |
|  | Appears miserable, unhappy tearful or distressed |
| **Hyperactivity**  (0.38 – 0.59) | Restless, runs about or jumps up and down. Doesn’t keep still |
|  | Squirmy, fidgety |
|  | Has poor concentration, or short attention span |
| **Peer problems** | Not much liked by other children |
| (0.23 – 0.41) | Tends to do things on own, rather solitary |
|  | Gets on well with other children |
| **Prosocial behaviour** | Considerate of other people’s feelings |
| (0.24 – 0.58) | Volunteers to help around the house or garden |
|  | Will try to help someone who has been hurt |
|  | Kind to younger children |
|  | Shares out treats with friends |

### **Table S4: SDQ scale items**

| **Trait measured** | **Items used (ages 4 and 7 years)** |
| --- | --- |
| **Conduct** | Often has temper tantrums or hot tempers |
|  | Generally obedient, usually does what adults request |
|  | Often fights with other children or bullies them |
|  | Often lies or cheats |
|  | Steals from home, school or elsewhere |
| **Emotional problems** | Often complains of headaches, stomach-aches or sickness |
|  | Many worries, often seems worried |
|  | Often unhappy, down-hearted or tearful |
|  | Many fears, easily scared |
| **Hyperactivity** | Restless, overactive, cannot stay still for long |
|  | Constantly fidgeting or squirming |
|  | Easily distracted, concentration wanders |
|  | Thinks things out before acting |
|  | Sees tasks through to the end, good attention span |
| **Peer problems** | Rather solitary, tends to play alone |
|  | Has at least one good friend |
|  | Generally liked by other children |
|  | Picked on or bullied by other children |
|  | Gets on better with adults than with other children |
| **Prosocial behaviour** | Considerate of other people’s feelings |
|  | Shares readily with other children (treats, toys, pencils etc.) |
|  | Helpful if someone is hurt, upset or feeling ill |
|  | Kind to younger children |
|  | Often volunteers to help others (parents, teachers, other children) |

## **Model comparisons**

### **Table S5: Language**

| Model 1 | Model 2 | Test | AIC 1 | AIC 2 | Chi Sq | *P* |
| --- | --- | --- | --- | --- | --- | --- |
| Language_score ~ (1\|randomfamid) + (1\|randomfamid:randomid) | Language_score ~ (1\|randomfamid) + (1+age\|randomfamid:randomid) | Random slopes | 14822 | 14590 | 235.75 | <.001*** |
| Language_score ~ (1\|randomfamid) + (1+age\|randomfamid:randomid) | Language_score ~ ses + sex + sibnum + zygos + (1\|randomfamid) + (1+age\|randomfamid:randomid) | Covariates | 14590 | 14436 | 162.47 | <.001 *** |
| Language_score ~ ses + sex + sibnum + zygos + (1\|randomfamid) + (1+age\|randomfamid:randomid) | Language_score ~ twinship + age + ses + sex + sibnum + zygos + (1\|randomfamid) + (1+age\|randomfamid:randomid) | Fixed effects | 14436 | 14425 | 14.863 | <.001 *** |
| Language_score ~ twinship + age + ses + sex + sibnum + zygos + (1\|randomfamid) + (1+age\|randomfamid:randomid) | **Language_score ~ twinship * age + ses + sex + sibnum + zygos + (1\|randomfamid) + (1+age\|randomfamid:randomid)** | **Interaction** | **14425** | **14385** | **41.712** | **<.001 ***** |

Note: Bold font indicates model used for final analysis. *** = *p*<.001

### **Table S6: Cognition**

| Model 1 | Model 2 | Test | AIC 1 | AIC 2 | Chi Sq | *P* |
| --- | --- | --- | --- | --- | --- | --- |
| Cognition_score ~ (1\|randomfamid) + (1\|randomfamid:randomid) | Cognition_score ~ (1\|randomfamid) + (1+age\|randomfamid:randomid) | Random slopes | 12540 | 12374 | 170.74 | <.001 *** |
| Cognition_score ~ (1\|randomfamid) + (1+age\|randomfamid:randomid) | Cognition_score ~ ses + sex + sibnum + zygos + (1\|randomfamid) + (1+age\|randomfamid:randomid) | Covariates | 12374 | 12242 | 139.65 | <.001 *** |
| Cognition_score ~ ses + sex + sibnum + zygos + (1\|randomfamid) + (1+age\|randomfamid:randomid) | **Cognition_score ~ twinship + age + ses + sex + sibnum + zygos + (1\|randomfamid) + (1+age\|randomfamid:randomid)** | **Fixed effects** | **12242** | **12140** | **105.81** | **<.001 ***** |
| Cognition_score ~ twinship + age + ses + sex + sibnum + zygos + (1\|randomfamid) + (1+age\|randomfamid:randomid) | Cognition_score ~ twinship * age + ses + sex + sibnum + zygos + (1\|randomfamid) + (1+age\|randomfamid:randomid) | Interaction | 12140 | 12140 | 1.69 | 0.19 |

Note: Bold font indicates model used for final analysis. *** = *p*<.001

### **Table S7: Conduct Problems**

| Model 1 | Model 2 | Test | AIC 1 | AIC 2 | Chi Sq | *P* |
| --- | --- | --- | --- | --- | --- | --- |
| Conduct_score ~ (1\|randomfamid) + (1\|randomfamid:randomid) | Conduct_score ~ (1\|randomfamid) + (1+age\|randomfamid:randomid) | Random slopes | 17921 | 17888 | 36.47 | <.001*** |
| Conduct_score ~ (1\|randomfamid) + (1+age\|randomfamid:randomid) | Conduct_score ~ ses + sex + sibnum + zygos + (1\|randomfamid) + (1+age\|randomfamid:randomid) | Covariates | 17888 | 17782 | 114.41 | <.001 *** |
| Conduct_score ~ ses + sex + sibnum + zygos + (1\|randomfamid) + (1+age\|randomfamid:randomid) | **Conduct_score ~ twinship + age + ses + sex + sibnum + zygos + (1\|randomfamid) + (1+age\|randomfamid:randomid)** | **Fixed effects** | **17782** | **17724** | **61.98** | **<.001***** |
| Conduct_score ~ twinship + age + ses + sex + sibnum + zygos + (1\|randomfamid) + (1+age\|randomfamid:randomid) | Conduct_score ~ twinship * age + ses + sex + sibnum + zygos + (1\|randomfamid) + (1+age\|randomfamid:randomid) | Interaction | 17724 | 17726 | 0.05 | 0.82 |

Note: Bold font indicates model used for final analysis. *** = *p*<.001

### **Table S8: Emotional Problems**

| Model 1 | Model 2 | Test | AIC 1 | AIC 2 | Chi Sq | *P* |
| --- | --- | --- | --- | --- | --- | --- |
| Emotion_score ~ (1\|randomfamid) + (1\|randomfamid:randomid) | Emotion_score ~ (1\|randomfamid) + (1+age\|randomfamid:randomid) | Random slopes | 18408 | 18376 | 36.77 | <.001 *** |
| Emotion_score ~ (1\|randomfamid) + (1+age\|randomfamid:randomid) | Emotion_score ~ ses + sex + sibnum + zygos + (1\|randomfamid) + (1+age\|randomfamid:randomid) | Covariates | 18376 | 18352 | 31.31 | <.001*** |
| Emotion_score ~ ses + sex + sibnum + zygos + (1\|randomfamid) + (1+age\|randomfamid:randomid) | **Emotion_score ~ twinship + age + ses + sex + sibnum + zygos + (1\|randomfamid) + (1+age\|randomfamid:randomid)** | **Fixed effects** | **18352** | **18111** | **245.49** | **<.001***** |
| Emotion_score ~ twinship + age + ses + sex + sibnum + zygos + (1\|randomfamid) + (1+age\|randomfamid:randomid) | Emotion_score ~ twinship * age + ses + sex + sibnum + zygos + (1\|randomfamid) + (1+age\|randomfamid:randomid) | Interaction | 18111 | 18111 | 1.86 | 0.17 |

Note: Bold font indicates model used for final analysis. *** = *p*<.001

### **Table S9: Hyperactivity**

| Model 1 | Model 2 | Test | AIC 1 | AIC 2 | Chi Sq | *P* |
| --- | --- | --- | --- | --- | --- | --- |
| Hyperactivity_score ~ (1\|randomfamid) + (1\|randomfamid:randomid) | Hyperactivity _score ~ (1\|randomfamid) + (1+age\|randomfamid:randomid) | Random slopes | 17799 | 17727 | 76.43 | <.001*** |
| Hyperactivity _score ~ (1\|randomfamid) + (1+age\|randomfamid:randomid) | Hyperactivity_score ~ ses + sex + sibnum + zygos + (1\|randomfamid) + (1+age\|randomfamid:randomid) | Covariates | 17727 | 17586 | 148.78 | <.001*** |
| Hyperactivity _score ~ ses + sex + sibnum + zygos + (1\|randomfamid) + (1+age\|randomfamid:randomid) | Hyperactivity_score ~ twinship + age + ses + sex + sibnum + zygos + (1\|randomfamid) + (1+age\|randomfamid:randomid) | Fixed effects | 17586 | 17510 | 80.12 | <.001*** |
| Hyperactivity_score ~ twinship + age + ses + sex + sibnum + zygos + (1\|randomfamid) + (1+age\|randomfamid:randomid) | **Hyperactivity_score ~ twinship * age + ses + sex + sibnum + zygos + (1\|randomfamid) + (1+age\|randomfamid:randomid)** | **Interaction** | **17510** | **17505** | **6.89** | **0.01**** |

Note: Bold font indicates model used for final analysis. *** = *p*<.001, ** = *p*<.01

### **Table S10: Peer Problems**

| Model 1 | Model 2 | Test | AIC 1 | AIC 2 | Chi Sq | *P* |
| --- | --- | --- | --- | --- | --- | --- |
| Peer_problems_score ~ (1\|randomfamid) + (1\|randomfamid:randomid) | Peer_problems_score ~ (1\|randomfamid) + (1+age\|randomfamid:randomid) | Random slopes | 18324 | 18287 | 40.68 | <.001*** |
| Peer_problems_score ~ (1\|randomfamid) + (1+age\|randomfamid:randomid) | Peer_problems_score ~ ses + sex + sibnum + zygos + (1\|randomfamid) + (1+age\|randomfamid:randomid) | Covariates | 18287 | 18201 | 93.71 | <.001*** |
| Peer_problems_score ~ ses + sex + sibnum + zygos + (1\|randomfamid) + (1+age\|randomfamid:randomid) | Peer_problems_score ~ twinship + age + ses + sex + sibnum + zygos + (1\|randomfamid) + (1+age\|randomfamid:randomid) | Fixed effects | 18201 | 18144 | 61.10 | <.001*** |
| Peer_problems_score ~ twinship + age + ses + sex + sibnum + zygos + (1\|randomfamid) + (1+age\|randomfamid:randomid) | **Peer_problems_score ~ twinship * age + ses + sex + sibnum + zygos + (1\|randomfamid) + (1+age\|randomfamid:randomid)** | **Interaction** | **18144** | **18141** | **5.53** | **0.02*** |

Note: Bold font indicates model used for final analysis. *** = *p*<.001, *=*p*<.05

### **Table S11: Prosocial Behaviour**

| Model 1 | Model 2 | Test | AIC 1 | AIC 2 | Chi Sq | *P* |
| --- | --- | --- | --- | --- | --- | --- |
| prosocial_score ~ (1\|randomfamid) + (1\|randomfamid:randomid) | prosocial_score ~ (1\|randomfamid) + (1+age\|randomfamid:randomid) | Random slopes | 17994 | 17916 | 81.47 | <.001*** |
| prosocial_score ~ (1\|randomfamid) + (1+age\|randomfamid:randomid) | prosocial_score ~ ses + sex + sibnum + zygos + (1\|randomfamid) + (1+age\|randomfamid:randomid) | Covariates | 17916 | 17802 | 122.68 | <.001*** |
| prosocial_score ~ ses + sex + sibnum + zygos + (1\|randomfamid) + (1+age\|randomfamid:randomid) | **prosocial_score ~ twinship + age + ses + sex + sibnum + zygos + (1\|randomfamid) + (1+age\|randomfamid:randomid)** | **Fixed effects** | **17802** | **17784** | **21.26** | **<.001***** |
| prosocial_score ~ twinship + age + ses + sex + sibnum + zygos + (1\|randomfamid) + (1+age\|randomfamid:randomid) | prosocial_score ~ twinship * age + ses + sex + sibnum + zygos + (1\|randomfamid) + (1+age\|randomfamid:randomid) | Interaction | 17784 | 17783 | 3.29 | 0.07 |

Note: Bold font indicates model used for final analysis. *** = *p*<.001

## **Table S12: Final model estimates**

| Model | *B* | SE | 95% CI |
| --- | --- | --- | --- |
| Language ~ twinship | -0.42 | 0.06 | -0.53 - -0.31 |
| + age | -0.07 | 0.01 | -0.09 - -0.05 |
| + SES | 0.21 | 0.02 | 0.17 - 0.25 |
| + sex | -0.14 | 0.02 | -0.19 - -0.10 |
| + zygosity | 0.10 | 0.04 | 0.02 - 0.19 |
| + household children | -0.04 | 0.02 | -0.07 - -0.01 |
| * age | 0.09 | 0.01 | 0.06 - 0.12 |
| Cognition ~ twinship | -0.17 | 0.02 | -0.20 - -0.14 |
| + age | 0.01 | 0.00 | 0.00 - 0.02 |
| + SES | 0.07 | 0.01 | 0.05 - 0.10 |
| + sex | -0.19 | 0.02 | -0.23 - -0.16 |
| + household children | -0.02 | 0.01 | -0.05 - 0.00 |
| + zygosity | 0.02 | 0.03 | -0.04 – 0.08 |
| Conduct ~ twinship | 0.21 | 0.03 | 0.16 - 0.26 |
| + age | -0.01 | 0.01 | -0.02 - 0.00 |
| + SES | -0.19 | 0.02 | -0.23 - -0.15 |
| + sex | 0.14 | 0.03 | 0.08 - 0.19 |
| + household children | 0.04 | 0.02 | 0.01 - 0.08 |
| + zygosity | 0.06 | 0.04 | -0.03 - 0.15 |
| Emotion ~ twinship | 0.42 | 0.03 | 0.37 - 0.47 |
| + age | -0.02 | 0.01 | -0.03 - -0.01 |
| + SES | -0.10 | 0.02 | -0.14 - -0.06 |
| + sex | -0.03 | 0.03 | -0.09 - 0.02 |
| + household children | 0.02 | 0.02 | -0.01 - 0.06 |
| + zygosity | 0.04 | 0.04 | -0.04 - 0.12 |
| Hyperactivity ~ twinship | 0.14 | 0.06 | 0.01 - 0.26 |
| + age | -0.04 | 0.01 | -0.07 - -0.02 |
| + SES | -0.18 | 0.02 | -0.22 - -0.14 |
| + sex | 0.27 | 0.03 | 0.21 - 0.33 |
| + household children | 0.01 | 0.02 | -0.03 - 0.04 |
| + zygosity | -0.03 | 0.04 | -0.11 – 0.05 |
| *age | 0.04 | 0.01 | 0.01 – 0.07 |
| Peer problems ~ twinship | 0.07 | 0.06 | -0.05 - 0.20 |
| + age | -0.04 | 0.01 | -0.07 - -0.02 |
| + SES | -0.12 | 0.02 | -0.16 - -0.08 |
| + sex | 0.14 | 0.03 | 0.08 - 0.20 |
| + household children | 0.06 | 0.02 | 0.03 - 0.10 |
| + zygosity | 0.10 | 0.04 | 0.02 - 0.18 |
| *age | 0.04 | 0.02 | 0.01 - 0.07 |
| Prosocial ~ twinship | -0.12 | 0.03 | -0.17 - -0.07 |
| + age | 0.00 | 0.01 | -0.01 - 0.01 |
| + SES | -0.01 | 0.02 | -0.05 - 0.03 |
| + sex | -0.32 | 0.03 | -0.37 - -0.26 |
| + household children | -0.01 | 0.02 | -0.05 - 0.03 |
| + zygosity | 0.06 | 0.05 | -0.03 – 0.15 |

Note: *B* = unstandardised model estimate, SE = standard error, 95%CI = 95% confidence intervals.

## **Table S13: Post hoc pairwise comparisons**

| Measure | Age | Est | SE | 95%CI | *p* | *d* |
| --- | --- | --- | --- | --- | --- | --- |
| Language | 2 | 0.24 | 0.03 | 0.18 - 0.30 | <.001*** | 0.41 |
|  | 3 | 0.15 | 0.02 | 0.11 - 0.20 | <.001*** | 0.26 |
|  | 4 | 0.06 | 0.02 | 0.02 - 0.10 | .002** | 0.12 |
|  | 7 | -0.21 | 0.05 | -0.30 - -0.11 | <.001*** | -0.35 |
| Hyperactivity | 2 | -0.21 | 0.04 | -0.29 - -0.13 | <.001*** | -0.33 |
|  | 3 | -0.25 | 0.03 | -0.31 - -0.19 | <.001*** | -0.39 |
|  | 4 | -0.29 | 0.03 | -0.35 - -0.23 | <.001*** | -0.45 |
|  | 7 | -0.40 | 0.06 | -0.51 - -0.29 | <.001*** | -0.62 |
| Peer problems | 2 | -0.15 | 0.04 | -0.22 - -0.07 | <.001*** | -0.19 |
|  | 3 | -0.18 | 0.03 | -0.24 - -0.13 | <.001*** | -0.24 |
|  | 4 | -0.22 | 0.03 | -0.28 - -0.17 | <.001*** | -0.29 |
|  | 7 | -0.33 | 0.06 | -0.44 - -0.22 | <.001*** | -0.43 |
| Cognition | - | 0.17 | 0.02 | 0.14 - 0.20 | <.001*** | 0.34 |
| Conduct | - | -0.21 | 0.03 | -0.26 - -0.16 | <.001*** | -0.30 |
| Emotional problems | - | -0.42 | 0.03 | -0.47 - -0.37 | <.001*** | -0.55 |
| Prosocial behaviour | - | 0.12 | 0.03 | 0.07 - 0.17 | <.001** | 0.17 |

Note: Est = unstandardised estimate, SE = standard error, 95%CI = 95% confidence intervals, *d* = Cohen’s d. Positive estimates indicate singletons outperform twins. A Tukey HSD correction was used to control for multiple comparisons. **= *p*<.01, ***= *p*<.001

## Table S14: Sex match/mismatch model estimates

| Model | *B* | SE | 95% CI |
| --- | --- | --- | --- |
| Language ~ twinship | -0.44 | 0.06 | -0.57 - -0.31 |
| + age | -0.06 | 0.01 | -0.09 - -0.04 |
| + SES | 0.24 | 0.03 | 0.19 - 0.29 |
| + sex | -0.17 | 0.03 | -0.23 - -0.11 |
| + zygosity | 0.08 | 0.05 | 0.02 - 0.17 |
| + household children | -0.04 | 0.02 | -0.08 - 0.00 |
| * age | 0.09 | 0.02 | 0.06 - 0.12 |
| + sex match/mismatch | **-0.03** | **0.05** | **-0.13 – 0.06** |
| Cognition ~ twinship | -0.19 | 0.02 | -0.23 - -0.15 |
| + age | 0.01 | 0.01 | 0.00 - 0.02 |
| + SES | 0.09 | 0.02 | 0.05 - 0.12 |
| + sex | -0.18 | 0.02 | -0.23 - -0.14 |
| + household children | -0.01 | 0.01 | -0.04 - 0.02 |
| + zygosity | 0.01 | 0.03 | -0.06 – 0.08 |
| + sex match/mismatch | **-0.03** | **0.03** | **-0.10 – 0.04** |
| Conduct ~ twinship | 0.22 | 0.03 | 0.16 - 0.28 |
| + age | -0.01 | 0.01 | -0.02 - 0.00 |
| + SES | -0.19 | 0.03 | -0.24 - -0.14 |
| + sex | 0.09 | 0.04 | 0.02 - 0.16 |
| + household children | 0.05 | 0.02 | 0.01 - 0.09 |
| + zygosity | 0.08 | 0.05 | -0.02 - 0.18 |
| + sex match/mismatch | **-0.03** | **0.05** | **-0.12 – 0.07** |
| Emotion ~ twinship | 0.43 | 0.03 | 0.37 - 0.49 |
| + age | -0.02 | 0.01 | -0.03 - -0.01 |
| + SES | -0.08 | 0.02 | -0.13 - -0.04 |
| + sex | -0.07 | 0.04 | -0.14 - 0.00 |
| + household children | 0.04 | 0.02 | 0.00 - 0.08 |
| + zygosity | 0.08 | 0.05 | -0.01 - 0.18 |
| + sex match/mismatch | **0.01** | **0.05** | **-0.08 – 0.11** |
| Hyperactivity ~ twinship | 0.12 | 0.07 | -0.02 - 0.26 |
| + age | -0.04 | 0.02 | -0.07 - -0.01 |
| + SES | -0.16 | 0.03 | -0.21 - -0.11 |
| + sex | 0.18 | 0.04 | 0.11 - 0.26 |
| + household children | 0.02 | 0.02 | -0.02 - 0.06 |
| + zygosity | -0.03 | 0.05 | -0.12 – 0.07 |
| *age | 0.04 | 0.02 | 0.01 – 0.07 |
| + sex match/mismatch | **-0.04** | **0.05** | **-0.13 – 0.05** |
| Peer problems ~ twinship | 0.07 | 0.08 | -0.08 - 0.22 |
| + age | -0.04 | 0.02 | -0.08 - -0.01 |
| + SES | -0.12 | 0.02 | -0.17 - -0.08 |
| + sex | 0.13 | 0.04 | 0.06 - 0.20 |
| + household children | 0.06 | 0.02 | 0.02 - 0.10 |
| + zygosity | 0.13 | 0.05 | 0.04 - 0.22 |
| *age | 0.03 | 0.02 | 0.00 - 0.07 |
| + sex match/mismatch | **-0.05** | **0.05** | **-0.14 – 0.04** |
| Prosocial ~ twinship | -0.12 | 0.03 | -0.17 - -0.06 |
| + age | 0.00 | 0.01 | -0.01 - 0.01 |
| + SES | 0.00 | 0.03 | -0.06 - 0.05 |
| + sex | -0.29 | 0.04 | -0.36 - -0.22 |
| + household children | 0.00 | 0.02 | -0.05 - 0.04 |
| + zygosity | 0.04 | 0.05 | -0.07 – 0.14 |
| + sex match/mismatch | **0.07** | **0.05** | **-0.03 – 0.18** |

Note: *B* = unstandardised model estimate, SE = standard error, 95%CI = 95% confidence intervals.
